# CagA-positive *Helicobacter pylori* may promote and aggravate scrub typhus

**DOI:** 10.3389/fmicb.2024.1351784

**Published:** 2024-01-17

**Authors:** Qiuying Du, Houyang Zeng, Xianwu Pang, Jianyu Cao, Bo Xie, Chunyi Long, Liudan Liang, Fenglian Deng, Meijin Huang, Li Li, Fengyan Huang, Xinli Liu, Yanling Hu, Jiannan Lv

**Affiliations:** ^1^Department of Infectious Diseases, Affiliated Hospital of Youjiang Medical University for Nationalities, Baise, Guangxi, China; ^2^Department of Infectious Diseases, Beihai People's Hospital, Beihai, Guangxi, China; ^3^Guangxi Zhuang Autonomous Region Center for Disease Control and Prevention, Nanning, Guangxi, China; ^4^Institute of Life Sciences, Guangxi Medical University, Nanning, Guangxi, China; ^5^Center for Genomic and Personalized Medicine, Guangxi key Laboratory for Genomic and Personalized Medicine, Guangxi Collaborative Innovation Center for Genomic and Personalized Medicine, Guangxi Medical University, Nanning, Guangxi, China

**Keywords:** *Helicobacter pylori*, scrub typhus, Th1 immune response, CagA gene, *Orientia tsutsugamushi*

## Abstract

*Helicobacter pylori* (*H. pylori*) infection may alter the host’s resistance to *tsutsugamushi* disease pathogens through the Th1 immune response, leading to potential synergistic pathogenic effects. A total of 117 scrub typhus cases at Beihai People’s Hospital and affiliated hospitals of Youjiang University for Nationalities and Medical Sciences were studied from January to December 2022, alongside 130 healthy individuals forming the control group. All participants underwent serum *H. pylori* antibody testing. The prevalence of *H. pylori* infection was significantly higher among scrub typhus patients (89.7%) compared to healthy individuals (54.6%) (*p* < 0.05). Moreover, type I *H. pylori* infection was notably more prevalent in scrub typhus cases (67.5%) compared to healthy individuals (30%) (*p* < 0.05). Multifactorial analysis demonstrated type I *H. pylori* infection as an independent risk factor for scrub typhus (adjusted odds ratio: 2.407, 95% confidence interval: 1.249–4.64, *p* = 0.009). Among scrub typhus patients with multiple organ damage, the prevalence of type I *H. pylori* infection was significantly higher (50.6%) than type II *H. pylori* infection (15.4%) (*χ*2 = 4.735, *p* = 0.030). These results highlight a higher incidence of *H. pylori* infection in scrub typhus patients compared to the healthy population. Additionally, type I *H. pylori* strain emerged as an independent risk factor for scrub typhus development. Moreover, individuals infected with type I *H. pylori* are more susceptible to multiple organ damage. These findings suggest a potential role of *H. pylori* carrying the CagA gene in promoting and exacerbating scrub typhus.

## Introduction

Scrub typhus is a contagious disease caused by *Orientia tsutsugamushi*, which was transmitted to humans through the bite of larval trombiculid mites. It poses a significant public health threat and is a major concern for travelers worldwide. In the Asia-Pacific region alone, over one billion people are at risk of *Orientia tsutsugamushi* infection (Taylor et al., 2015). China, particularly the Guangxi Zhuang Autonomous Region in southern China, was considered a highly endemic area for scrub typhus. The primary clinical manifestations of scrub typhus include fever, rash, eschar, and enlarged lymph nodes. If left untreated, the disease can lead to various complications such as toxic hepatitis, myocarditis, pneumonia, meningoencephalitis, acute renal failure, and disseminated intravascular coagulation. In severe cases, septic shock and multiple organ failure can occur, resulting in a high fatality rate ranging from 30 to 70% (Taylor et al., 2015). *Orientia tsutsugamushi* primarily targets the systemic vascular endothelial system and parasitizes host endothelial cells, macrophages, monocytes, and dendritic cells (Kang et al., 2017). While Th1-mediated cellular immunity plays a crucial role in the body’s defense against this pathogen, an excessive Th1-type immune response can also cause tissue damage (Anwesha and Smita, 2021; James et al., 2021). Studies have demonstrated that serum levels of inflammatory cytokines such as IL-6, IL-8, IL-10, MCP-1, and TNF are strongly associated with the severity of scrub typhus (Inthawong et al., 2023).

*Helicobacter pylori* is a gram-negative bacterium that colonizes the mucosal surface of the stomach and establishes a lifelong, chronic infection. In recent years, there has been growing interest in the relationship between *H. pylori* and extragastric diseases. Research has shown that *H. pylori* was closely associated with infectious diseases caused by pathogens such as HIV, HCV, and brucella (Furusyo et al., 2011; Afrasiabian et al., 2017; Guo et al., 2021). Additionally, *H. pylori* may have a protective role against tuberculosis, allergic diseases, asthma, shigellosis, and other diarrheal diseases (Blaser et al., 2008; Perry et al., 2010; Cohen et al., 2012). Upon colonization, *H. pylori* activates NF-κB transcription factors, leading to the secretion of chemokines like IL-8 by epithelial cells and other cells. This attracts and activates inflammatory cells, promotes the production of Th1-type cytokines such as IFN-γ, and contributes to gastric inflammation (Bridge and Merrell, 2013). The virulence protein CagA, expressed by *H. pylori*, plays a significant role in *H. pylori*-host interactions. Individuals who test positive for CagA may experience more severe tissue inflammation and elicit stronger cytokine responses (Bridge and Merrell, 2013). Furthermore, *H. pylori* induces the secretion of IL-10 through the regulatory T cell (Treg) network, which helps control local inflammation and maintain immune homeostasis. This immune response is particularly pronounced in infections with cagA+/vacA s1m1 strains, and co-infection with *H. pylori* and helminths can also increase susceptibility to tuberculosis due to dysregulated immune responses (Bustamante-Rengifo et al., 2021). These immune mechanisms contribute to the complex interactions between *H. pylori* and various infectious diseases.

Infection with *Orientia tsutsugamushi* in humans can manifest with a wide range of clinical symptoms, varying from mild (asymptomatic) to severe and potentially life-threatening conditions (Park et al., 2021; Devamani et al., 2022). The underlying mechanisms for these variations are not fully understood, but it was believed that factors such as the strain of *Orientia tsutsugamushi* and the immune status of the host may play a role (Elliott et al., 2019). Therefore, it is important to investigate whether certain infectious factors that influence host immunity have a synergistic effect on the development of scrub typhus. Both *H. pylori* infection (Fulian et al., 2009) and scrub typhus (Seung-Ji et al., 2016) exhibit a tendency towards Th1 immune responses and share similarities in their immune mechanisms. It was speculated that *H. pylori* infection may alter the body’s susceptibility to *Orientia tsutsugamushi* and potentially have a synergistic pathogenic effect. Hence, this study aims to explore the correlation between *H. pylori*, its various subtypes, and scrub typhus.

## Materials and methods

### Sample calculation

The sample size was determined using a power analysis for an independent samples *t*-test. Based on an expected effect size (Cohen’s d) of 0.4, a significance level (*α*) of 0.05, and a desired power (1−*β*) of 0.80, the calculated sample size was approximately 85 participants per group. This sample size estimation was conducted to ensure adequate statistical power to detect differences between groups for the specified effect size and level of significance.

### Sample information

Total of 117 patients who were clinically diagnosed with scrub typhus and admitted to Beihai People’s Hospital and the Affiliated Hospital of Youjiang Medical University for Nationalities between January and December 2022 were included in this study. The cohort consisted of 51 males and 66 females, with an average age of 56 years old. According to the number of complications, they were further divided into two groups: 72 cases in the <3 kinds of complications group and 45 cases in the ≥3 kinds of (defined as multiple organ damage) group. The diagnostic criteria for scrub typhus, as per the 4th edition of Practice of Infectious Diseases (Wang and Li, 2017), were as follows: ① History of field activity in the past 3 weeks; ② Fever, ③ Presence of eschar or ulcer; ④ Hepatosplenomegaly or lymph node enlargement; ⑤ Weil-Felix reaction ≥1:160. For this study, the clinical diagnostic criteria required patients to meet all three of the following criteria: ①, ②, and ③ at the same time as the diagnostic basis. 130 cases of healthy subjects matched for gender and age from the above two hospitals at the same peroid were selected as the control group, including 59 males and 71 females, with an average age of 54 years old. Prior approval for the study was obtained from the ethical review committees of Beihai People’s Hospital and the Affiliated Hospital of Youjiang Medical University for Nationalities. All patients were provided with detailed information about the study, including potential risks and benefits, and gave their voluntary consent by signing informed consent forms. Based on *H. pylori* test results, the 117 patients diagnosed with scrub typhus were divided into two groups: 105 *H. pylori*-positive and 12 negative patients. Table 1 presents a comparison of the general data between these two cohorts.

**Table 1:** Comparison of general characteristics between *H. pylori* positive and *H. pylori* negative patients with Scrub typhus disease.

Characteristics	Scrub typhus patients (*n* = 117)		*χ*^2^/*t*-value	*p-*value
	*H. pylori* (+) (*n* = 105)	*H. pylori* (−) (*n* = 12)		
Male, [*n* (%)]	45 (42.9)	6 (50.0)	0.084	0.771
Age (±s), y	56.1 ± 14.89	61.25 ± 15.99	1.981	0.262
Residency, [*n* (%)]			6.026	0.014
Rural	65 (61.9)	3 (25.0)		
Urban	40 (38.1)	9 (75.0)		
complication [*n* (%)]			3.808	0.051
≥ 3 kinds of complications	44 (41.9%)	1 (8.3%)		
< 3 kinds of complications	61 (58.1%)	11 (91.7%)		
*H. pylori* [*n* (%)]				
Type I	79 (75.2)	0 (0)		
Type II	26 (24.8)	0 (0)		

### Subtyping detection of *H. pylori*

Peripheral blood samples were collected from 117 *tsutsugamushi* patients and 130 healthy subjects, and serum *H. pylori* antibodies were analyzed using Western immunoblotting. This technique, provided by Shenzhen Burroughs Biological Company, allowed for the detection of specific antibodies related to *H. pylori* subtypes. The immunoblotting method enabled the identification of *H. pylori* cytotoxin-associated gene A (CagA) through the presence of bands at 128 kD and 116 kD, vacuolating toxin A (VacA) with bands at 95 kD and 91 kD, urease B (ureB) with a band at 66 kD, and urease A (ureA) with a band at 30 kD. Type I (virulent/carrying the CagA gene) *H. pylori* infection was determined by the presence of CagA and/or VacA bands, type II (non-virulent/not carrying the CagA gene) *H. pylori* infection was indicated by the presence of urease antibodies only, without CagA and VacA bands, and negative results for all three antibodies were classified as negative for *H. pylori* antibodies. The experiments were conducted strictly following the instructions, using reagents purchased from Shenzhen Brault Biological Company with production batch number 161216.

### Statistical analysis

The data were analyzed using SPSS 19.0 statistical software. Count data were presented as frequency and percentage and compared using the χ^2^ test. Measurement data were expressed as mean ± standard deviation (x ± s) and compared using the t-test. Logistic regression analysis was conducted to identify influencing factors. Statistical significance was determined at a value of p of less than 0.05. The data were statistically analyzed using SPSS 19.0 statistical software. Enumeration data were presented as the number of cases and percentages and were compared using the *χ*^2^ test. Measurement data were presented as mean ± standard deviation (x ± s) and were compared using the *t*-test. Logistic regression analysis was used to identify influencing factors. A *p*-value less than 0.05 was considered statistically significant.

## Results

### Analysis of the status of *H. pylori* infection in patients with scrub typhus

Among the 117 patients with scrub typhus, 105 tested positive for *H. pylori*, resulting in an infection rate of 89.7% (105/117). This included 79 cases (67.5%) of type I *H. pylori* infection and 26 cases (22.2%) of type II *H. pylori* infection. Among the 130 healthy subjects, 71 cases were positive for *H. pylori*, resulting in an infection rate of 54.6% (71/130). This included 39 cases (30.0%) of type I *H. pylori* infection and 32 cases (24.6%) of type II *H. pylori* infection. The prevalence of *H. pylori* infection and type I *H. pylori* infection was higher in patients with *tsutsugamushi* disease compared to healthy individuals (*χ*^2^ = 6.225, *p* = 0.013; *χ*^2^ = 12.314, *p* < 0.001), respectively. The difference was not statistically significant when comparing the prevalence of type II *H. pylori* infection (*χ*^2^ = 0.122, *p* = 0.727). The details were showed in Table 2.

**Table 2:** *H. pylori* infection status in patients with *tsutsugamushi* disease.

	Scrub typhus patients (*n* = 117)	Healthy subjects (*n* = 130)
*H. pylori*, *n* (%)	105 (89.7)	71 (54.6)
Type I, *n* (%)	79 (67.5)	39 (30.0)
Type II, *n* (%)	26 (22.2)	32 (24.6)

### Multifactorial regression analysis of the relationship between different types of *H. pylori* infection and scrub typhus

Multiple regression analysis as used to further explore the relationship between different types of *H. pylori* infection and scrub typhus. After adjusting for gender, age and place of residence, the results revealed that type I *H. pylori* infection was an independent risk factor for the occurrence of scrub typhus (2.407; 1.249–4.64; 0.009). The details were showed in Table 3.

**Table 3:** Multivariate regression analysis of the relationship between *H. pylori* infection and *tsutsugamushi* disease.

Variable	*β* coefficient	Standard error	Wald *χ*^2^	*p*-value	OR (95% Confidence interval)
Sex (male/female)	−0.087	0.319	0.075	0.785	0.916 (0.49,1.714)
Residency (Rural/Urban)	−0.208	0.332	0.393	0.531	0.812 (0.423,1.557)
Age (≥65/<65)	0.158	0.355	0.199	0.656	1.172 (0.584,2.351)
*H. pylori* (type I/type II)	0.878	0.335	6.88	0.009	2.407 (1.249,4.64)

### The correlation between different types of *H. pylori* infection and the presence of multiple organ damage in patients with scrub typhus

**Figure 1:**
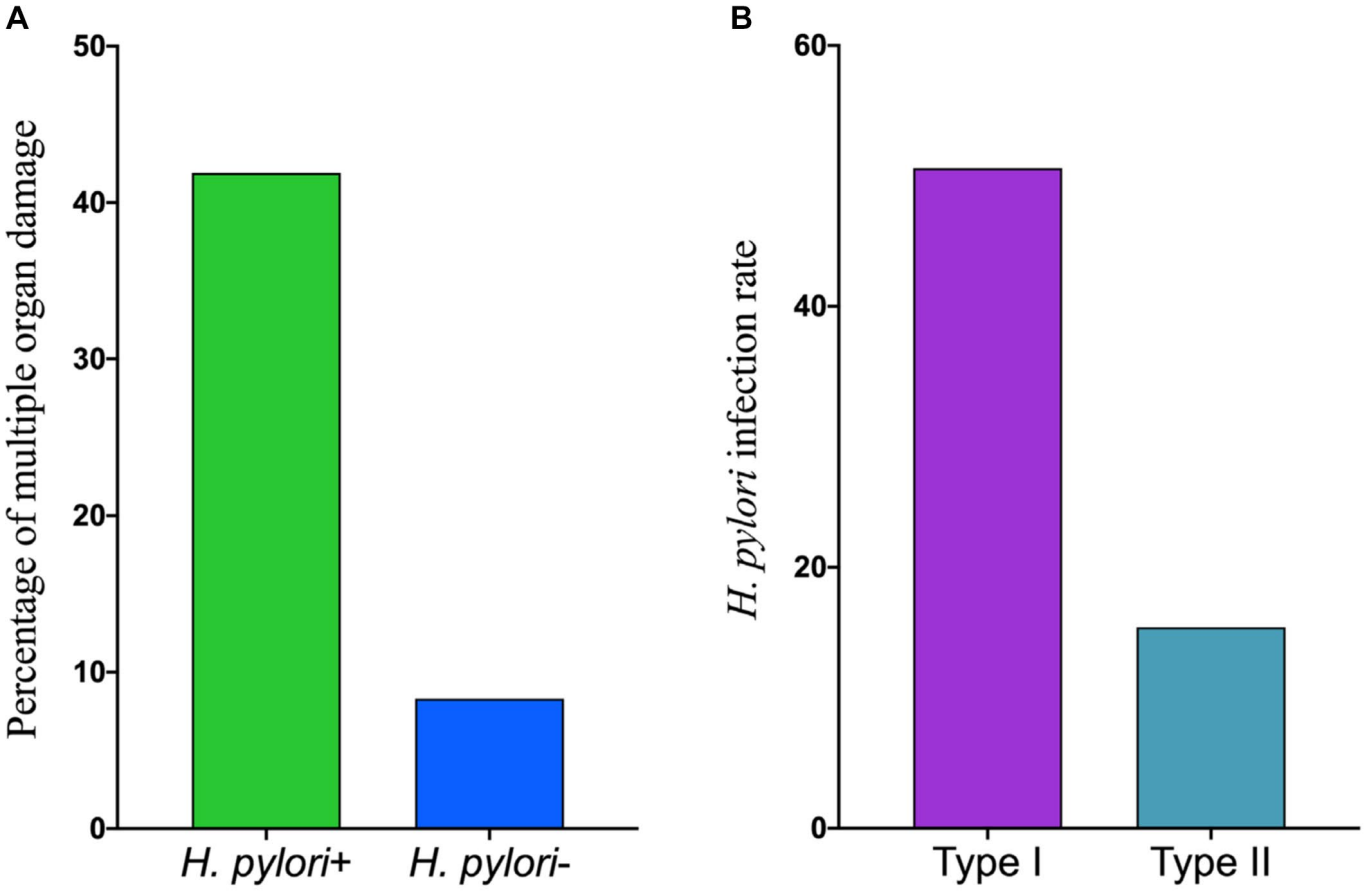
The correlation between different types of *H. pylori* infection and the presence of mutiple organ damage in scrub typhus patients. **(A)** The percentage of mutiple organ damage in scrub typhus patients. **(B)** The infection rate of different *H. pylori* type.

Among the 117 patients diagnosed with scrub typhus, 45 individuals (38.5%) were found to have more than three comorbidities, which were defined as multiple organ damage. Further analysis was conducted to examine the different subtypes of *H. pylori* infection among patients with scrub typhus who experienced multiple organ damage. Out of the 45 patients suffering from scrub typhus with multiple organ damage, the prevalence of *H. pylori* positivity was observed to be 41.9% (44/105), whereas the *H. pylori* negativity accounted for 8.3% (1/12). These proportions did not exhibit any statistically significant differences (x^2^ = 1.858, *p* = 0.173). The proportion of *H. pylori* infections with type I was 50.6% (40/79), which was significantly higher than that with type II *H. pylori* infection rate of 15.4% (4/26), (x^2^ = 4.735, *p* = 0.030). For detailed information, please refer to Figure 1.

## Discussion

*Helicobacter pylori* infection is commonly acquired during childhood and if left untreated, it can persist for a lifetime (Frenck and Clemens, 2003). In contrast, scrub typhus is an acute infectious disease caused by opportunistic infection of the host with *Orientia tsutsugamushi*. The status of *H. pylori* infection in scrub typhus patients and whether *H. pylori* infection has an impact on the occurrence and development of scrub typhus has not been reported. Epidemiological surveys have shown that the infection rate of *H. pylori* infection is related to social economic situation, living conditions, sanitary conditions and living habits, occupation and quality of drinking water (Xu Wenting and Chengfu, 2022). The prevalence of scrub typhus is also found in specific populations (e.g., military personnel or plantation workers) and agricultural staff in townships (Devamani et al., 2022). To avoid any influence of environmental and occupational factors on the study results, we strictly matched the regional distribution, age, and gender of the *Tsutsugamushi* patients and the healthy medical examination population.

This study has made a novel discovery, demonstrating that the prevalence of *H. pylori* infection was significantly higher in scrub typhus patients compared to healthy subjects (89.7% vs. 53.8%, *p* < 0.05). This suggests that individuals infected with *Orientia tsutsugamushi* are more likely to develop typical scrub typhus if they already have an existing *H. pylori* infection. This finding may help explain why not all *Orientia tsutsugamushi* infected hosts develop *tsutsugamushi* (Park et al., 2021). The exact mechanism underlying this phenomenon is not yet fully understood. However, it is believed that dysregulation of cytokine levels, Th1/Th2 cytokine imbalance, and T lymphocyte apoptosis during acute infection with *H. tsutsugamushi* play a role in pathogenesis, indicating that virulence of *Orientia tsutsugamushi* alone is not solely responsible, but host immune responses also contribute (Seung-Ji et al., 2016). *H. pylori* infection triggers activation of NF-xB transcription factors, leading to secretion of IL-8 and other chemokines by epithelial cells or other cells, which stimulate chemotaxis and activation of inflammatory cells, resulting in an inflammatory response (Bridge and Merrell, 2013). Cytokines induced by *H. pylori* infection act on tissues and organs throughout the body via blood circulation, potentially contributing to the typical clinical manifestations observed in scrub typhus patients with *H. pylori* infection due to the combined effect of Th1 cytokines.

It is notewothy that the effect of *H. pylori* infection on host immunity is multifaceted and consequently produces different clinical outcomes when co-infected with other pathogens. During *H. pylori* infection, neutrophils, B and T lymphocytes infiltrate in the gastric and duodenal mucosa, but they are unsuccessful in clearing the infection. This may be attributed to inadequate cytokine secretion and immune response down-regulation due to an increase in the number of regulatory T cells (Tregs) (Hu et al., 2009). Other studies have also shown an increase in the number of Tregs and a decrease in cytokine production during Helicobacter infection (Ismail et al., 2002; Wang et al., 2004; Park et al., 2007). Increased Tregs activity has been associated with the stability of infections such as leishmaniasis, malaria and tuberculosis, suggesting a possible association between the survival and persistence of some infectious agents and the immunosuppression of Tregs (Belkaid et al., 2002). This also partly explains why *H. pylori* may have a protective effect on tuberculosis, allergic diseases, asthma, shigellosis and other diarrhea diseases (Blaser et al., 2008; Perry et al., 2010; Cohen et al., 2012). Furthermore, *H. pylori* replicates and retains cytoplasmic MHC class II molecules within the autophagosome; it also hampers IL-12 production and triggers IL-10 secretion, thus hindering the antimicrobial Th1 response (Gorvel et al., 2014). Therefore, we infer that the robust antimicrobial Th1 response that ought to have been initiated in individuals infected with *H. pylori* at the onset of *Orientia tsutsugamushi* invasion might be attenuated; in other words, the host’s anti-infective immune response has been modified during chronic *H. pylori* infection, augmenting susceptibility to *tsutsugamushi*.

The vast majority of *H. pylori* infections in childhood are asymptomatic, but some individuals who are infected may still develop gastrointestinal or extragastrointestinal diseases. In addition to host genetic factors, the possible mechanism for this is closely associated with the virulence type of *H. pylori* strains, particularly the infection by CagA-positive strains (Bridge and Merrell, 2013). *H. pylori* possesses a range of virulence factors, including VacA, urease, CagA, heat shock proteins, and neutrophil activating proteins (Fulian et al., 2009). Among these, CagA stands out as one of the most crucial virulence factors. Strains of *H. pylori* that contain the CagA gene are known as high-virulence strains, as they express both VacA and CagA proteins. On the other hand, *H. pylori* strains without the CagA gene are classified as low-virulence strains, lacking expression of VacA and CagA proteins. In this study, immunoblotting was employed for the diagnosis of *H. pylori* infection through strain typing, allowing for an analysis of the correlation between different types of *H. pylori* infection and scrub typhus. The findings revealed a significantly higher prevalence of *H. pylori* infection in patients with scrub typhus type I (carrying the CagA gene) compared to healthy subjects (89.7% vs. 53.8%, *p* < 0.05), while the prevalence of *H. pylori* infection in type II (not carrying the CagA gene) was similar to that in healthy subjects and did not show any statistically significant difference (*p* > 0.05). Moreover, the multivariate regression analysis indicated that *H. pylori* infection of type I was an independent risk factor for scrub typhus. Furthermore, among patients afflicted with scrub typhus and experiencing multiple organ damage, the prevalence of type I *H. pylori* infection amounted to 50.6%, markedly surpassing the incidence of type II *H. pylori* infection at 15.4% (x2 = 4.735, *p* = 0.030). These findings suggests that the development of scrub typhus may be associated with the virulence factor of *H. pylori* containing the CagA gene. Possible mechanisms for this include: on the one hand, CagA is one of the main virulence proteins expressed by *H. pylori*, significantly increased mRNA expression of C-X-C chemokines in the gastric mucosa of CagA-positive individuals, and strains of *H. pylori* that produce VacA and CagA proteins cause more severe tissue inflammation and a more intense cytokine response. Additionally, CagA-positive strains were found to be potent inducers of IL-8 (Hu et al., 2009). Therefore, it is speculated that the Th1 response induced by *H. pylori* type I strain infection may be more intense. On the other hand, the cross immune reaction hypothesis of molecular simulation suggests that because the highly conserved protein sequences of *H. pylori* organisms have similar antigenic determinants to human vascular endothelium or glandular epithelium, certain antibodies such as CagA antibodies and HSP antibodies are induced to bind to human cells *in vivo* leading to cellular damage (Hu et al., 2009). Given that the primary pathological damage observed in scrub typhus is vascular endothelial damage, we speculate that the presence of CagA-producing strains of *H. pylori* infection may contribute to amplified vascular endothelial damage through a yet unknown pathway. This, in turn, could have a synergistic effect on the body’s inflammatory response. Clinically, this manifests as typical symptoms of scrub typhus, involvement of multiple organs, and potentially a predisposition towards severe disease.

There may be some limitations to this study. In this study, immunoblotting was employed to detect serum *H. pylori* antibodies and classify *H. pylori* infection. This method is known for its simplicity and high specificity and sensitivity. However, serological studies are prone to data bias, and we did not adopt two tests to minimize potential errors. Secondly, our data analysis was confined solely to serological examinations, thus neglecting the evaluation of other influential factors that could impact *H. pylori* infection, such as current infection status, household income, education level, and antibiotic usage.

## Conclusion

The prevalence of *H. pylori* infection was significantly greater in patients with scrub typhus than in the healthy population, and the type I *H. pylori* strain was an independent risk factor for scrub typhus, Moreover, individuals infected with type I *H. pylori* are more susceptible to experiencing multiple organ damage associated with scrub typhus, suggesting that *H. pylori* carrying the CagA gene may promote and exacerbate scrub typhus.

## Data availability statement

The original contributions presented in the study are included in the article/supplementary material, further inquiries can be directed to the corresponding author.

## Ethics statement

The studies involving humans were approved by the ethical review committees of Beihai People’s Hospital and the Affiliated Hospital of Youjiang Medical University for Nationalities. The studies were conducted in accordance with the local legislation and institutional requirements. The participants provided their written informed consent to participate in this study.

## Author contributions

QD: Writing – review & editing, Data curation, Methodology, Validation, Writing – original draft. HZ: Data curation, Validation, Writing – original draft, Writing – review & editing, Resources. XP: Data curation, Validation, Writing – original draft, Writing – review & editing, Methodology. JC: Data curation, Validation, Writing – review & editing. BX: Data curation, Writing – review & editing, Formal analysis, Methodology, Visualization. CL: Writing – review & editing, Resources, Validation. LiuL: Resources, Validation, Writing – review & editing. FD: Writing – review & editing, Data curation. MH: Writing – review & editing, Validation. LiL: Writing – review & editing, Resources. FH: Writing – review & editing. XL: Writing – review & editing. YH: Writing – review & editing, Conceptualization, Project administration, Supervision. JL: Supervision, Project administration, Supervision, Writing – review & editing, Funding acquisition.
